# Posteromedial elbow dislocation with associated lateral humeral condyle fracture in a child: a rare case report and literature review

**DOI:** 10.1093/jscr/rjag022

**Published:** 2026-01-27

**Authors:** Mishari Alanezi, Nouf Alabdulkarim, Mohammad Firas Serro, Shaker Mohammed Alamir

**Affiliations:** College of Medicine, King Saud University, PO Box 2925, Riyadh 11461, Saudi Arabia; Department of Orthopedic Surgery, College of Medicine, King Saud University, PO Box 2925, Riyadh 11461, Saudi Arabia; Department of Orthopedic Surgery, King Saud Medical City, PO Box 2925, Riyadh 11461, Saudi Arabia; Department of Orthopedic Surgery, King Saud Medical City, PO Box 2925, Riyadh 11461, Saudi Arabia

**Keywords:** posteromedial elbow dislocation, lateral condyle fracture, case report

## Abstract

Elbow dislocations are rare in children, comprising 3%–6% of pediatric elbow injuries, with posteromedial dislocations being particularly uncommon. We describe an 8-year-old boy who sustained a posteromedial elbow dislocation with an associated displaced lateral humeral condyle fracture after a fall. Although closed reduction restored joint alignment, the condylar fragment remained displaced and required fixation with two Kirschner wires. At 6 weeks, radiographs confirmed union, and the wires were removed. By 6 months, the patient had regained full, painless motion with a normal carrying angle and no growth disturbance. This rare injury pattern may be overlooked due to overlapping ossification centers, highlighting the importance of prompt diagnosis and appropriate surgical management.

## Introduction

Elbow dislocations in the pediatric population are uncommon, representing 3%–6% of pediatric elbow injuries [1]. Among these, posteromedial dislocations are particularly rare and even more exceptional when accompanied by a fracture of the lateral humeral condyle [2]. Although lateral condyle fractures (LCF) are the second most common distal humerus fracture in children [3], their coexistence with a posteromedial dislocation is extremely rare.

This injury likely results from axial loading with valgus stress on a partially flexed elbow, leading to lateral column failure and joint displacement [4]. Given the intra-articular nature of the fracture and instability after dislocation, early recognition and proper management are crucial to avoid complications such as nonunion, malalignment, and stiffness [5].

Clinically, this injury presents with severe pain, swelling, and restricted elbow motion, requiring prompt assessment [6]. Radiographs may be challenging to interpret due to subtle fractures and overlapping ossification centers, and computerized tomography (CT) may be necessary for accurate evaluation and treatment planning [7].

In this article, we describe a rare case of an 8-year-old boy with a posteromedial elbow dislocation and associated lateral humeral condyle fracture. Informed consent was obtained from his guardian.

## Case report

**Figure 1:**
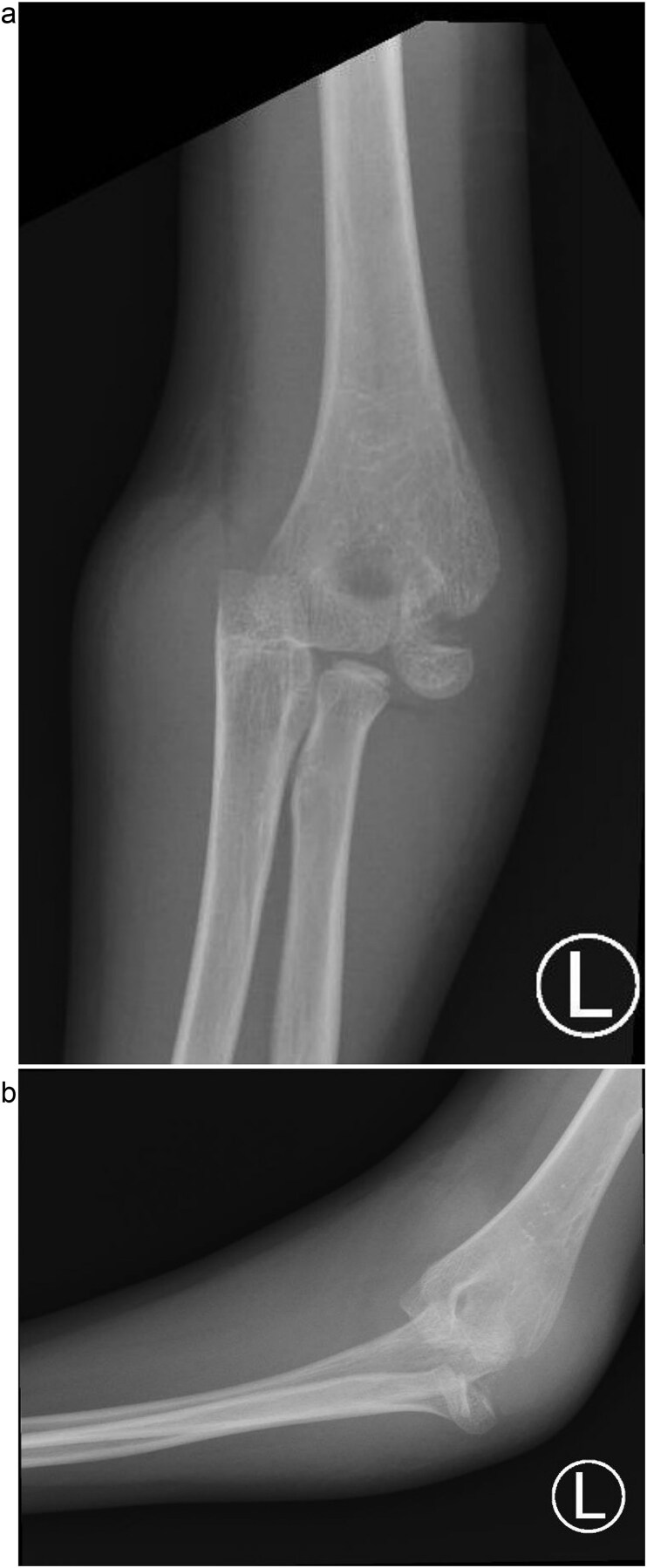
(a) Pre-reduction anteroposterior radiograph of the left elbow showing posteromedial dislocation of the ulnohumeral joint with displaced lateral humeral condyle fracture. (b) Lateral radiograph demonstrating posterior displacement of the olecranon and lateral condyle fragment.

An 8-year-old boy presented to the emergency department with severe left elbow pain and deformity after falling while playing football. He reported landing on his arm but could not recall the exact elbow position. Examination showed significant swelling, deformity, and lateral elbow tenderness. The skin was intact, distal pulses and capillary refill were normal, and neurological assessment confirmed intact radial, ulnar, median, and anterior interosseous nerve function. Radiographs in anteroposterior and lateral views, along with a 3D CT scan, demonstrated a posteromedial dislocation of the elbow associated with a displaced fracture of the lateral condyle of the humerus (Fig. 1a).

**Figure 2:**
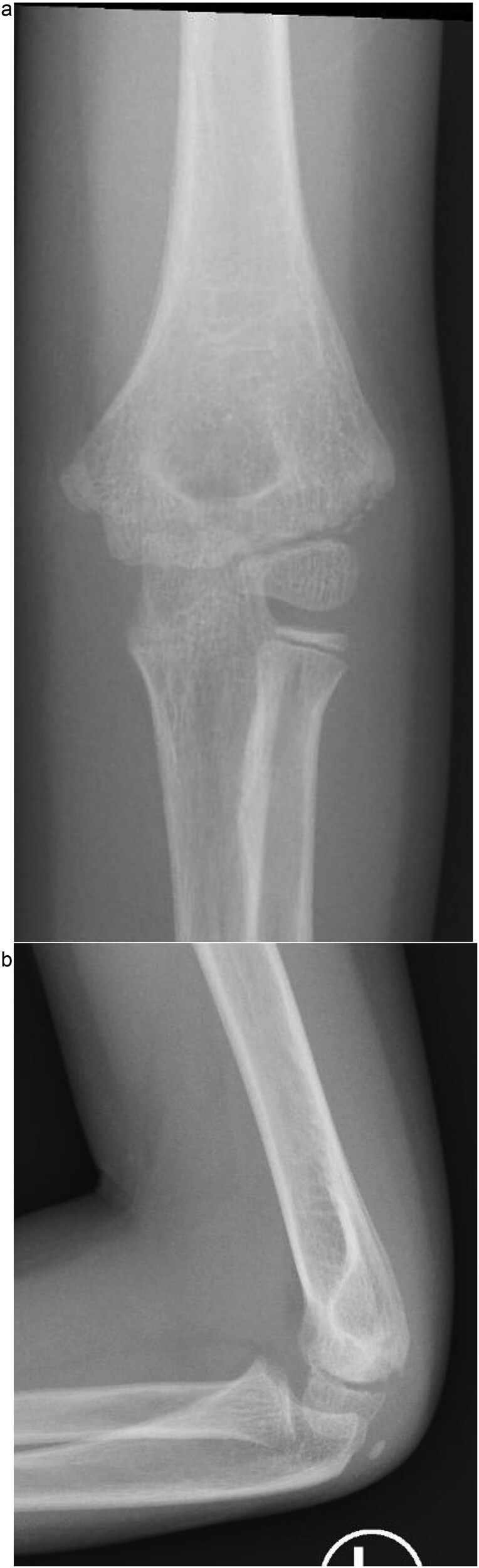
(a) Immediate post-reduction anteroposterior radiograph showing restored joint alignment with persistent lateral condyle displacement. (b) Lateral view confirming maintained reduction of the ulnohumeral joint and residual separation of the lateral condyle fragment.

**Figure 3:**
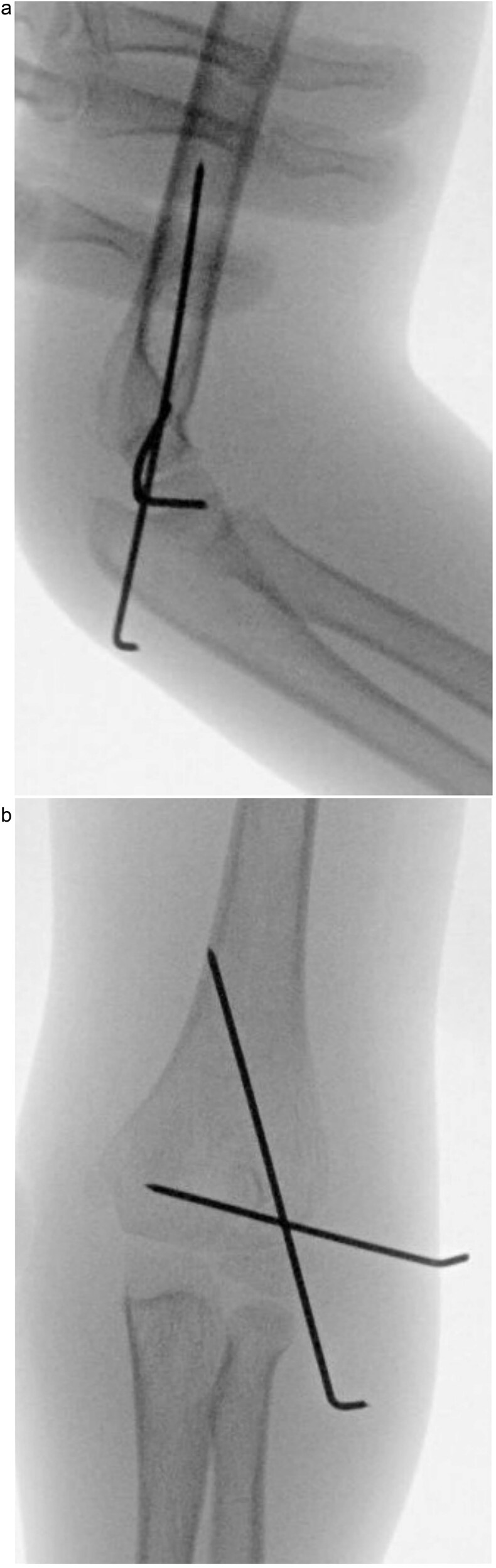
(a) Intraoperative fluoroscopic image following closed reduction and K-wire fixation showing anatomical alignment of the lateral condyle. (b) Arthrogram confirming a congruent articular surface and stable fixation of the fracture fragment.

**Figure 4:**
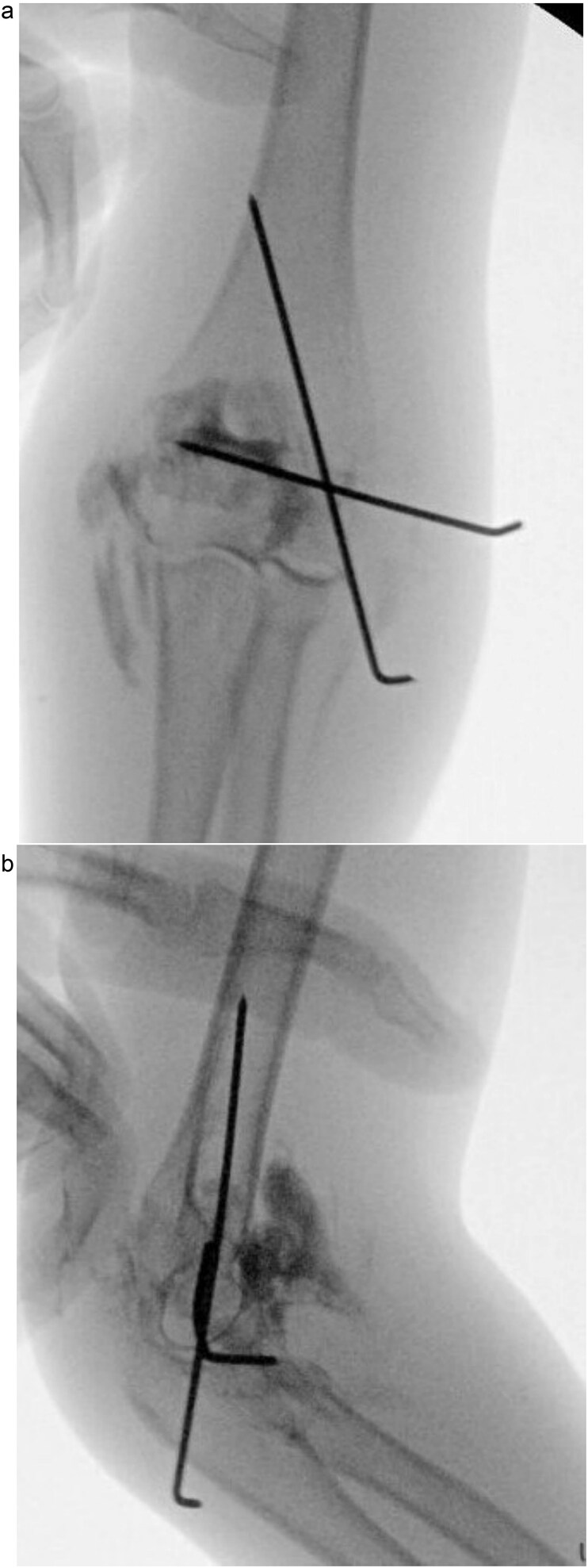
(a) Postoperative anteroposterior radiograph demonstrating stable fixation of the lateral condyle with two smooth Kirschner wires. (b) Lateral view showing proper wire placement and a well-aligned elbow maintained in a posterior slab splint.

Under procedural sedation, closed reduction of the elbow dislocation was performed, restoring joint alignment and stability. However, post-reduction imaging showed persistent displacement of the lateral condyle fragment (Fig. 2a). The patient was subsequently taken to the operating room, where closed reduction of the condylar fracture was carried out under general anesthesia. As post-reduction displacement was less than 2–4 mm with intact articular cartilage, fixation was performed using two smooth Kirschner wires under fluoroscopic guidance. An arthrogram confirmed joint congruity and cartilage integrity (Fig. 3a). A long-arm posterior slab was applied with the elbow in <90° of flexion to minimize the risk of compartment syndrome (Fig. 4a).

**Figure 5:**
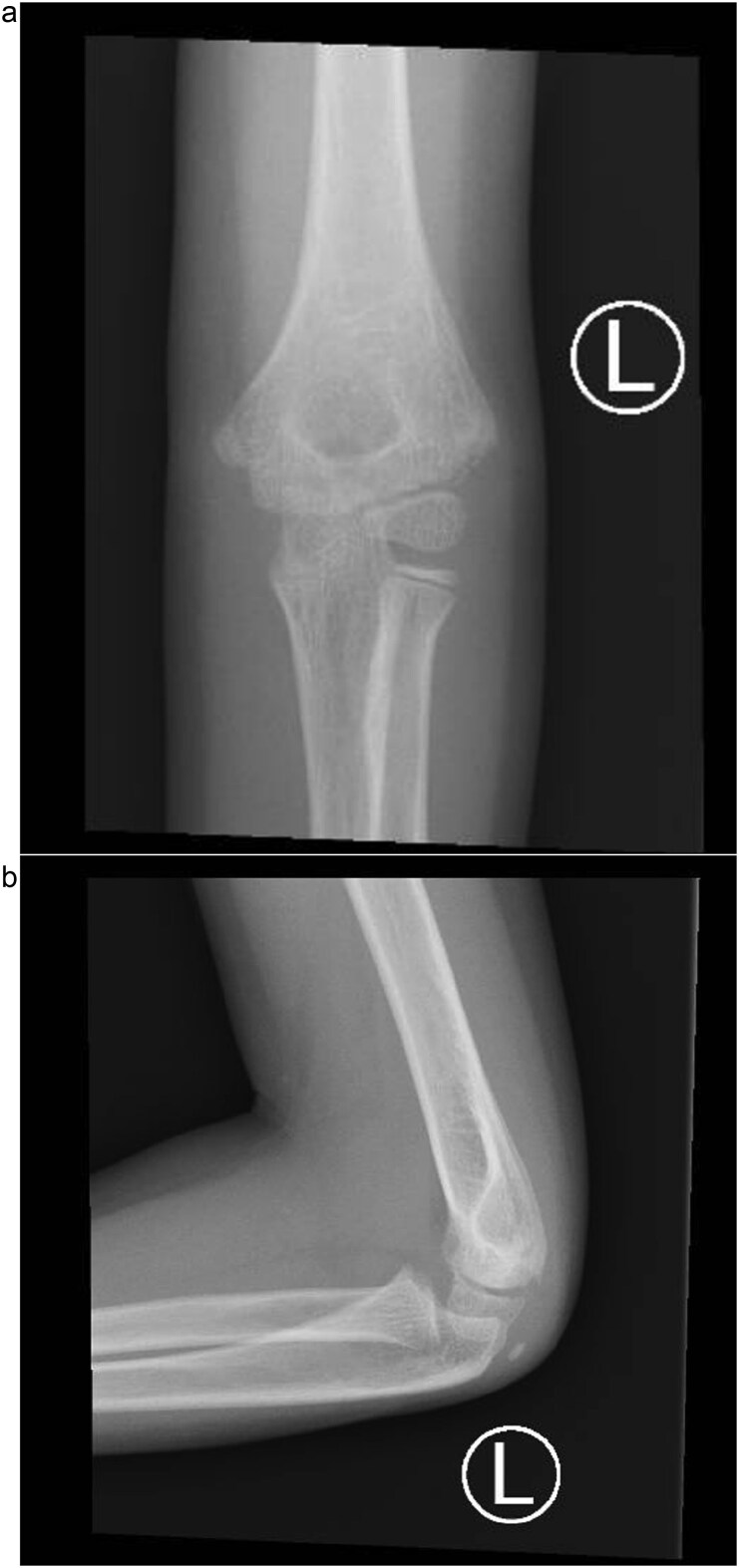
(a) Six-month follow-up anteroposterior radiograph showing complete union of the lateral condyle fracture after Kirschner wire removal. (b) Six-month lateral radiograph demonstrating full healing with restored joint alignment, maintained carrying angle, and no evidence of growth disturbance or deformity.

At the 3-week follow-up, dressing changes and radiographs showed satisfactory alignment and stable fixation. By 6 weeks, radiographs confirmed union of the lateral condyle, the Kirschner wires were removed, and mobilization began. At 3 months, the patient had painless motion from 10° to 120° with a stable joint. By 6 months, he achieved full range of motion and a normal carrying angle, with no radiographic signs of growth disturbance, avascular necrosis, or fishtail deformity (Fig. 5a).

## Discussion

Elbow dislocations in children are uncommon, and posteromedial types are especially rare, particularly when combined with a LCF [8, 9]. These injuries typically cause sudden, severe pain, swelling, and marked motion restriction, with the limb held in slight flexion. Prompt neurovascular assessment is essential due to the nearby brachial artery and median nerve [4].

LCFs are intra-articular fractures involving the capitellar ossification center and part of the trochlea. The Milch classification describes two patterns: Type I, where the fracture line passes lateral to the trochlea through the capitulotrochlear groove, sparing the trochlear articulation and usually preserving stability; and Type II, where the line extends into the trochlea, disrupting the articulation and causing greater instability. Although Type II fractures are more often associated with dislocations, this case shows that even a Milch Type I injury can occur with significant joint displacement when high-energy valgus and axial forces are involved.

Diagnosis is typically made using anteroposterior and lateral radiographs to confirm the dislocation and identify associated fractures. When plain films are unclear, CT or oblique views can help define fracture morphology and assist in surgical planning [5, 8].

Management requires prompt reduction of the dislocation and stabilization of the fracture. While isolated pediatric elbow dislocations can be treated with closed reduction and brief immobilization, displaced lateral condyle fractures typically need internal fixation to restore joint congruity. Early surgery improves outcomes and reduces the risks of nonunion, malunion, growth disturbance, and instability [9–11]. Fixation options include K-wires or cannulated screws, with screws offering greater stability and the potential for earlier mobilization and less postoperative stiffness [10, 12]. Postoperative immobilization is typically limited to 2–4 weeks before starting range-of-motion exercises. With timely diagnosis and appropriate surgical treatment, most children recover well, though long-term follow-up is important to detect any late complications [2, 9].

Complications of LCFs, with or without dislocation, include nonunion, malunion, premature physeal closure, avascular necrosis, stiffness, and instability [10]. Neurovascular injury occurs in 10%–20% of displaced pediatric elbow fractures, most often involving the anterior interosseous, median, or ulnar nerves; these are typically neurapraxias that recover but require close monitoring [11, 12]. Vascular compromise from brachial artery spasm, entrapment, or intimal injury can lead to limb ischemia or Volkmann contracture if unrecognized [13].

Given these risks, careful neurovascular assessment is essential before and after reduction. Motor function should be tested for the radial (wrist/finger extension), median (thumb opposition/flexion), anterior interosseous (‘OK’ sign), and ulnar nerves (finger abduction/adduction). Sensory testing should include the first dorsal web space (radial), the volar index fingertip (median), and the volar little fingertip (ulnar). Perfusion must be evaluated with capillary refill, skin temperature, and color, with Doppler ultrasound or pulse oximetry used when findings are unclear [3].

## Conclusion

This rare combination of posteromedial elbow dislocation and lateral humeral condyle fracture can be easily overlooked. Prompt reduction and stable fixation are key to restoring joint function and preventing growth complications.

## Data Availability

This study did not create new data; therefore, data sharing is not applicable.
